# Unusual localized gingival redness: a case report

**DOI:** 10.3389/froh.2023.1292332

**Published:** 2023-11-28

**Authors:** Swati Kumar, Nanditha Sujir, Anwesha Saha, Junaid Ahmed, Prem Bhushan

**Affiliations:** ^1^ Community Health Center, Angara, India; ^2^ Department of Oral Medicine and Radiology, Manipal College of Dental Sciences Mangalore, Manipal Academy of Higher Education, Manipal, India; ^3^ Department of Prosthodontics and Crown & Bridge, Awadh Dental College & Hospital, Jamshedpur, India

**Keywords:** gingival carcinoma, red lesion, early diagnosis, case report, oral cancer

## Abstract

Inflammation of the gingiva is one of the most common and routine findings in dental practice. These routine appearances of inflammatory gingivae can show peculiarity when associated with an underlying systemic condition or because of reactive, benign, or malignant pathologies. This case highlights minute clinical signs of the gingiva that deviate from the routine presentation and warrant further investigations. A 63-year-old woman presented with a chief complaint of severe pain in relation to the lower front teeth region for 1 month. Intraoral examination revealed a gingival lesion on the labial aspect of 41, 42, and 43, and an intraoral periapical radiograph showed mild bone loss. The lesion persisted despite oral prophylaxis, and a biopsy was advised. The final diagnosis was stage 1 gingival squamous cell carcinoma (GSCC). It is important to note that the non-descript presentation of GSCC in early stages often mimics benign traumatic or inflammatory lesions of the gingiva. Peculiar clinical features of GSCC of note include the lack of traditionally associated risk factors and localized red or ulcerative lesions with increased bleeding tendencies that do not respond to routine periodontal treatment within 2 weeks.

## 1. Introduction

Inflammation of the gingiva is one of the most common and routine findings in dental practice. Morphological changes associated with chronic gingivitis and periodontitis present as redness of the gingiva, enlargement, loss of stippling, bleeding on probing, etc. These routine appearances of inflammatory gingiva can show peculiarity when they are associated with an underlying systemic condition (pregnancy, diabetes, etc.) or when there are local pathologies that are reactive, benign, or malignant in nature (1). Dentists need to identify minute changes in clinical appearance that deviate from the routine inflammatory conditions or benign lesions to pursue further investigations. We present one such case of a common finding of a red gingival lesion with a concerning diagnostic outcome.

## 2. Case description

**Figure 1:**
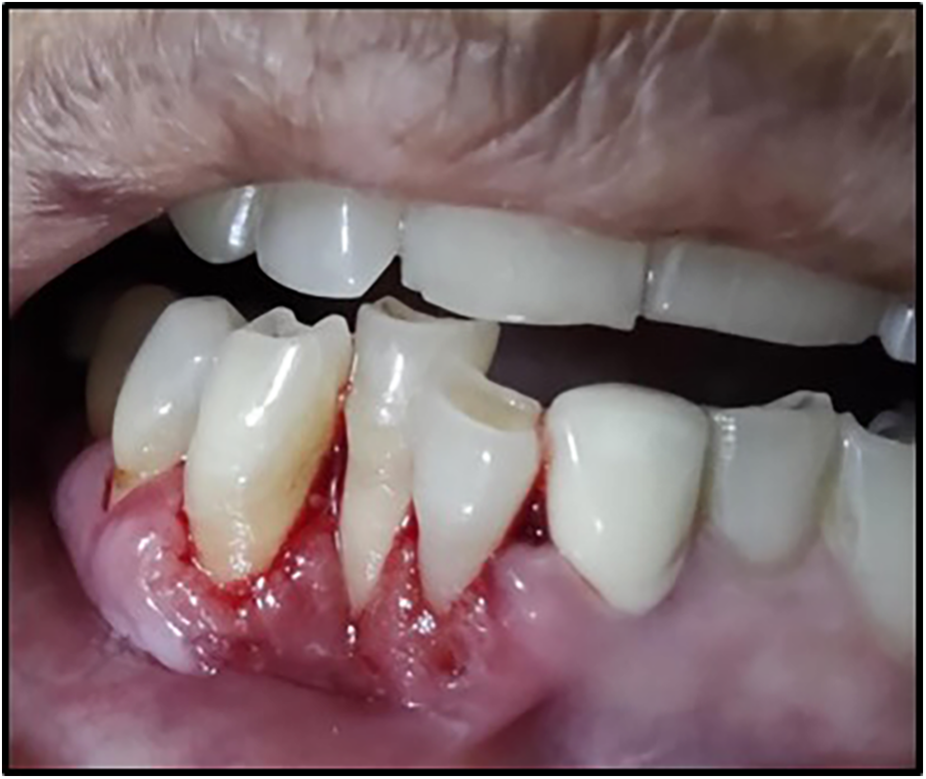
Red lesion of the gingiva.

A 63-year-old woman presented with a chief complaint of severe pain in the lower front teeth for 1 month (Figure 1). The patient reported severe pain upon mechanical stimulation in the region and avoided biting food. She reported occasional bleeding gums when brushing that area, which had persisted for 10 days. The patient had no history of any oral abusive habits, such as tobacco consumption or use of toothpicks. There was no history of trauma reported. Her medical history was positive for hypothyroidism and hypertension, for which the patient was on medication that controlled the conditions. Her family history was positive with her brother having undergone treatment for throat cancer. The patient did not report any previous consultation or use of home remedies for the treatment of the lesion.

**Figure 2:**
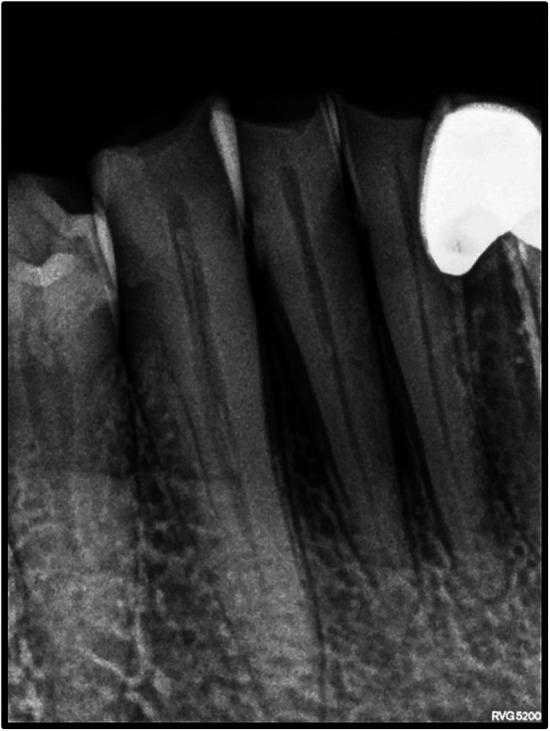
Intraoral periapical radiograph showing 41, 42, and 43 with mild horizontal bone loss.

On intraoral examination, there was a gingival lesion on the labial aspects of 41, 42, and 43, extending from the mesial aspect of 41 to the distal aspect of 43. The lesion involved marginal, interdental, and attached gingiva. The gingival lesion appeared to be erythematous, and the surface was irregular with erosions. The margins of the lesion were well-defined with raised edges. It was soft in consistency and tender on palpation. On periodontal examination, there was the presence of bleeding upon probing, with no exudation. There was a moderate deposition of calculi. There was an absence of mobility i.r.t 41, 42, and 43. There was no tenderness on lateral and vertical percussions on teeth i.r.t 41, 42, and 43. The rest of the oral mucosa had no significant findings. On extraoral palpation, submandibular lymph nodes on the right side were palpable, firm, non-tender, and mobile. An intraoral periapical radiograph (Figure 2) was taken and revealed mild horizontal interdental bone loss in the regions 42 and 43, which suggested chronic periodontitis.

### 2.1. Differential diagnosis

Considering the gingival lesion with a tendency for bleeding and the presence of interdental bone loss, chronic periodontitis exacerbated by an underlying systemic condition such as diabetes mellitus could be considered as a diagnosis in this case (2). The other commonly associated systemic conditions are vitamin C deficiency (3) and hormonal dysfunction (estrogen and progesterone) (4), which can lead to a friable and inflamed gingiva. However, such a systemic condition would cause a more generalized gingival involvement rather than a localized lesion.

Localized lesions involving gingivae may occur because of chronic trauma. Additionally, toothbrush injury, the use of toothpicks, abrasive dentifrices, and rare self-inflicted injuries may cause gingival erosions and ulcerations (5). However, a lack of relevant history and the overall irregular surface texture of the lesions made this an unlikely possibility. Erythroplakia and oral squamous cell carcinoma (OSCC) can present as localized lesions on the gingiva. Erythroplakia is a potentially malignant disorder that appears as a well-defined red lesion that, unlike leukoplakia, can appear as a mucosal depression. The surface of the lesion may be smooth, velvety, or granular. Erythroplakia is usually not associated with habits and could not be ruled out in this case (6). Considering a positive family history of malignancy, associated pain, and increased bleeding tendency of the lesion, OSCC can also be considered in this case (1, 7). Furthermore, apart from erythroplakia and OSCC, granulomatous conditions, such as tuberculosis (8), sarcoidosis (9), Wegner's granulomatosis (10), and deep fungal infections (11), can present similarly and can be ruled out with further investigation.

### 2.2. Management

Initially, a round of oral prophylaxis was carried out, and she was prescribed 0.2% chlorhexidine mouthwash. Additionally, she was advised to have a complete blood count and a random blood glucose test and recall after 2 weeks. The blood report was within normal range.

**Figure 3:**
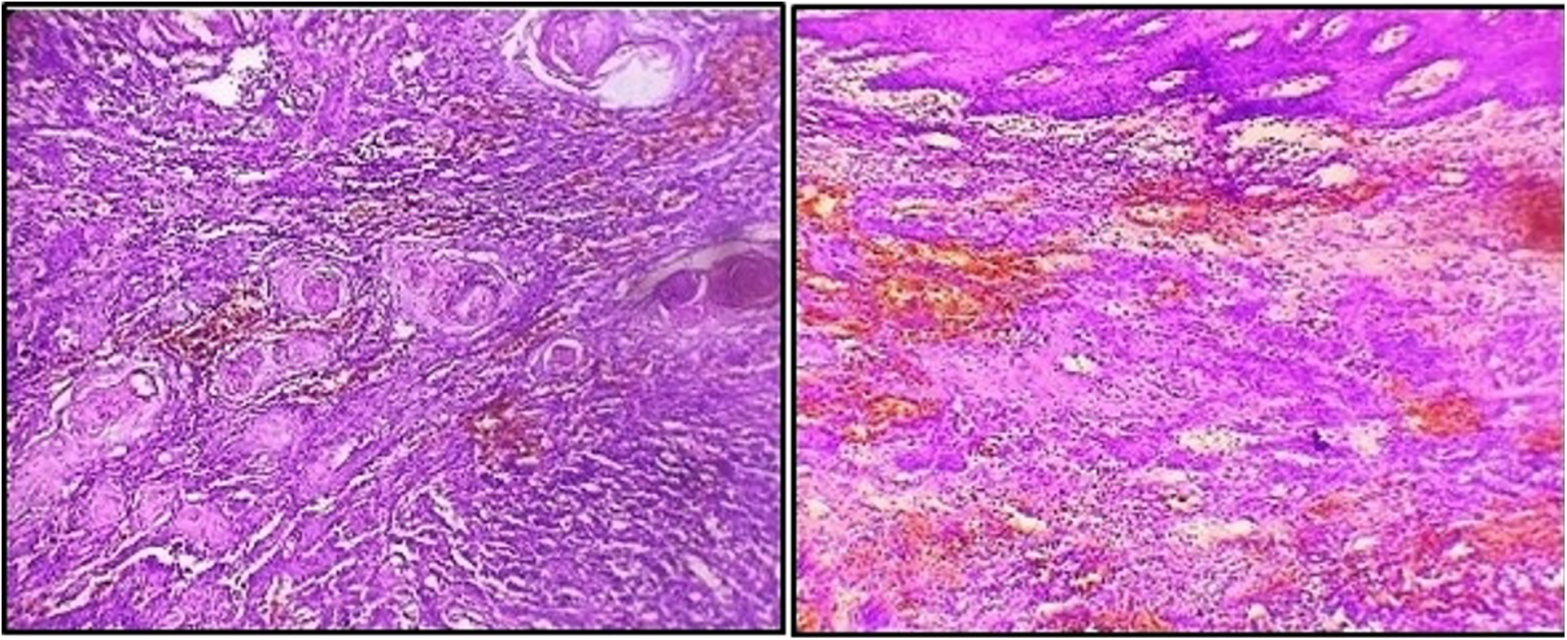
Photomicrograph showing squamous cell carcinoma features.

As the lesion persisted along with bleeding tendency during the follow-up visit, an incisional biopsy was performed. A histopathological report revealed a squamous epithelium overlying the connective tissue with tumors irregularly proliferating in the form of sheets and islands in the connective tissue stroma. Additionally, keratin pearls were evident within the tumor islands. Squamous tumor islands showed dysplastic features such as hyperchromatism, pleomorphism, an altered nuclear-cytoplasmic ratio, individual cell keratinization, and a few mitoses. The connective tissue stroma comprised bundles of collagen fibers with spindle-shaped fibroblasts, chronic inflammatory infiltrate, and endothelial-lined blood vessels with RBCs (Figure 3). Thus, the final diagnosis was well-differentiated OSCC.

**Figure 4:**
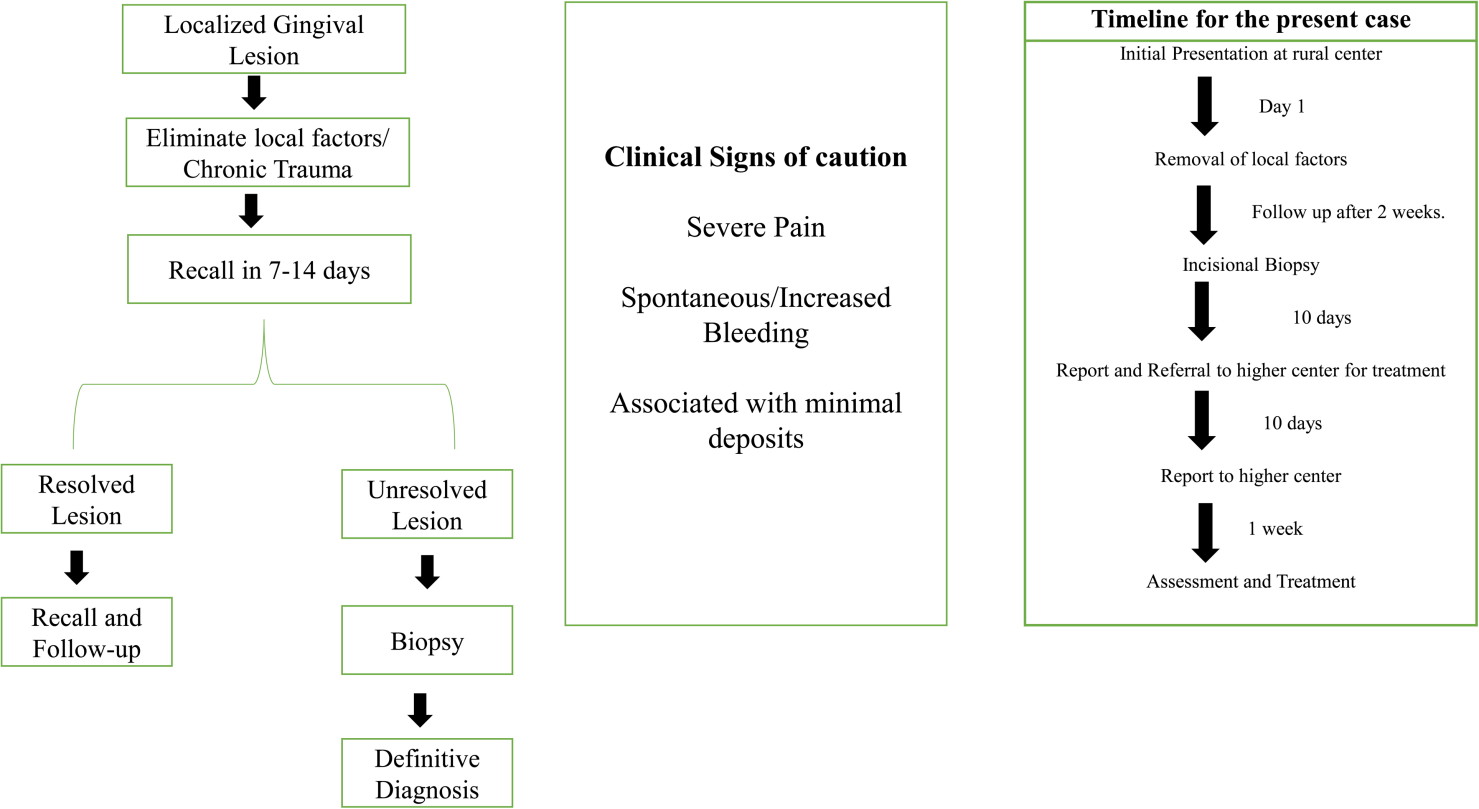
A guide to identify and manage suspicious red lesions of the gingiva.

The patient was referred to a higher center for surgical intervention. Further investigations, such as contrast CT, were performed, and the patient was diagnosed with gingival squamous cell carcinoma (GSCC) Stage 1 (T1, N0, M X). The patient underwent surgery and radiotherapy and is currently in follow-up. Figure 4 shows the timeline from the episode through the administration of care.

## 3. Discussion and conclusions

Oral cancer is a broad term that includes various malignant conditions involving the oral tissues. It is the sixth most common cancer in the world and OSCC accounts for 90% of all oral cancers (12). South Asian countries, particularly India, show the highest prevalence of OSCC. This is largely related to the widespread use of tobacco, betel quid, and alcohol, which are established risk factors (13). Approximately 10% of all OSCCs are known to be caused by the malignant transformation of potentially malignant disorders (12, 14), many of which are habit associated. In recent times, there has been an increase in the prevalence of oral cancer among populations that have no habit history, especially among younger individuals (15). The majority of oral cancers, especially in countries such as India, are diagnosed in the advanced stages. The treatment for the advanced stages is aggressive, which results in severe morbidity and poor health-related quality of life. The outcomes of treatment are poor, as 5-year survival rates for advanced stages are approximately 20%. Thus, early diagnosis and prompt treatment are essential for favorable patient outcomes. This case highlights several atypical features that need to be considered to identify OSCC during the initial stages and ensure prompt treatment.

In the present case, the patient did not report any deleterious habits that are usually associated with oral cancer. There are several risk factors associated with non-habit-associated malignancies (Figure 4). Although these risk factors are not well established, unlike tobacco and alcohol, their role in oral cancer has been reported in a few studies or case reports. Several authors have shown an increased risk of OSCC in first-degree relatives of patients previously diagnosed with malignancy (16). Genetic changes that are linked to an increased incidence of OSCC include TP53 mutations and CDKN2A inactivation, the expression of certain proto-oncogenes, and frequent copy number alterations (17, 18). Consumption of hot spicy food, presence of sharp teeth, poor oral hygiene, systemic inflammation, and conditions causing prolonged irritation are considered risk factors for oral cancer. However, these factors lack sufficient scientific evidence to establish a causal relationship (19, 20). Specific microorganisms, such as *Treponema pallidum* (syphilis), *Candida*, and human papillomavirus (HPV), have been associated with an increased risk of malignancy (12, 19). HPV has been isolated in approximately 23.5% of oral cancers (20) and sexual practices have been considered contributory to the higher incidence of HPV (21). Environmental carcinogens, such as ultraviolet rays, can contribute as risk factors. Dietary factors, such as iron deficiency and a poor diet with a lack of micronutrients and antioxidants, have been implicated in the pathogenesis of oral cancer (22). It should be noted that the causal relationship with oral cancer related to the abovementioned factors needs more clinical and experimental evidence. Unusual oral lesions coupled with the presence of risk factors should warrant further investigation. In the present case, the patient's immediate relative had been diagnosed with throat cancer; however, genetic testing was not feasible.

The most common sites of OSCC are the tongue and the floor of the mouth, followed by the soft palate, gingiva, and buccal mucosa. OSCC of the gingiva is normally painless and its incidence ranges from 2 to 27% (23, 24). Gender predilection is ambiguous with respect to GSCC, with some studies reporting female predilection (25) and others reporting male predilection (26). Mandibular involvement is usually more common than the maxilla. Additionally, GCSS is generally associated with non-traditional risk factors and is found in non-smokers and non-drinkers (1, 27). It is an insidious disease that is present without the typical clinical features attributable to malignancy and is frequently misdiagnosed as a periodontal inflammatory lesion. In such cases, presentations that could indicate malignancy include erythema with non-healing ulcers and red lesions with a granular surface texture that bleed profusely (1). It usually begins with the keratinized mucosa, quickly progressing to involve the underlying bone structure with ensuing tooth mobility (28). As the thickness of the attached gingiva is 2–3 mm, gingival carcinomas tend to involve the bone at early stages and can spread easily (29, 30). Dental extractions are usually carried out in cases that are misdiagnosed as periodontitis. Dental extractions lead to bone invasion and further spread, worsening the prognosis of the patient (31). It is essential to evaluate bony involvement in GSCC cases. Radiographic features that help distinguish malignant involvement as opposed to bone loss related to periodontitis include irregular bone loss with an associated moth-eaten appearance, non-uniform widening of the PDL space, and the absence of sclerosis of trabeculae that is associated with bone loss secondary to periodontitis. Thus, clinical and radiographic features should be carefully examined to diagnose gingival carcinoma at an early stage. This case had an insidious clinical presentation of redness of the gingiva that could be mistaken for a benign lesion. Additionally, there was an absence of typical malignant changes on the radiograph, with bone loss and accompanying sclerosis indicating chronic periodontitis. Furthermore, the lesion was associated with severe pain, which is unusual for a benign lesion. Although early stages of OSCC may be asymptomatic, pain is often an indicator of the transition of a potentially malignant lesion into a cancerous lesion and often the first presenting symptom in oral cancers. In this case, the severe pain and a lack of response of the lesion to local therapy was the main reason for suspicion and warranted further investigation (32, 33). A similar case of GSCC was reported by Brooks et al., in which ulcerations confined to the palatal marginal gingiva confined to the maxillary molars associated with profuse bleeding were observed.

A delay in the diagnosis of oral cancer can significantly impact the health care outcomes for the patient. Survival rates of patients negatively correlate with the stage of diagnosis. Treatment for advanced stages is aggressive and is related to a poor quality of life for survivors. Multiple factors, including patient-related delays, professional delays, and healthcare system-related delays, contribute to diagnostic delays (34). Patients may delay seeking medical care due to a lack of awareness of symptoms, the nature of the disease process, or phobias and anxiety related to cancer diagnosis. Additionally, socioeconomic constraints and cultural beliefs may contribute to a delay in diagnosis. There is poor public awareness related to oral health and the work scope of dental practitioners. Thus, patients do not opt for regular dental check-ups. Infrequent or lack of practice of regular dental check-ups excessively contributes to delays in diagnosis (35, 36). In our case, unusual pain and bleeding gums prompted the patient to seek medical care. A familial history of malignancy can also contribute to patient awareness of symptoms associated with malignancy.

Once the patient reports to a health care professional, it is the responsibility of the professional to aid in early diagnosis and treatment. Professional delays are known to occur due to a lack of awareness of oral cancer symptoms and misdiagnosis. Here, the dentist may not be the first point of contact of the patient, and patients may likely present initially to a non-dental health care professional. Thus, it is essential that all health care professionals and patients are aware of the signs and symptoms related to oral cancer (36). This is particularly true of GSCC with its innocuous presentation, as observed in the present case. Additionally, understaffing and resource constraints in the healthcare system can contribute to delays in the diagnosis and treatment of cancers. There is a wide disparity in the distribution of health care professionals between rural and urban areas. The at-risk population largely resides in rural areas with socioeconomic constraints. Our patient also lived in a rural area and reported to the community health center to seek a consultation with a dentist due to tooth-related pain. The presence of an oral medicine specialist at the rural hospital significantly contributed to early diagnosis in this case. Considering these factors, it is essential to raise awareness, train health care professionals, and strengthen healthcare systems to aid in the early diagnosis of oral cancer.

Currently, there are no screening protocols for individuals who do not exhibit traditional risk factors but may still be at risk for GSCC; routine oral examination is key to early diagnosis for such cases. Additionally, toluidine blue staining (TBS), oral cytology (OC), and light-based detection (LBD) devices, such as Velscope, ViziLite plus (37), and ultrasound (38), are known to aid the early diagnosis of oral cancer. However, the cost of implementing these chair-side investigations has hindered their widespread use. Indicators of poor prognosis in GSCC are the presence and extent of mandibular involvement and the advanced stage of diagnosis. The incidence of cervical lymph node metastasis associated with GSCC is higher in relation to the lesion on the posterior of the mandible (39). These points must be considered while evaluating the patient with GSCC.

This case report highlights the non-descript presentation of GSCC at early stages, which often mimics benign traumatic or inflammatory lesions of the gingiva. Peculiar clinical features of GSCC of note include the lack of traditional risk factors and localized red or ulcerative lesions with increased bleeding tendencies that do not respond to routine periodontal treatment of oral prophylaxis within 2 weeks. The affinity of GSCC to invade the bone and spread at an early stage makes early diagnosis and prompt treatment critical for improving therapeutic outcomes for the patient.

## Data Availability

The original contributions presented in the study are included in the article/Supplementary Material, further inquiries can be directed to the corresponding author.
